# Layered Double Hydroxides as an Intercalation System for Hydrophobic Molecules

**DOI:** 10.3390/nano13243145

**Published:** 2023-12-15

**Authors:** Lei Li, Anastasia Sevciuc, Patrick van Rijn

**Affiliations:** 1 Department of Biomedical Engineering-FB40, University of Groningen, University Medical Center Groningen, Groningen, A. Deusinglaan 1, 9713 AV Groningen, The Netherlands; 2 W.J. Kolff Institute for Biomedical Engineering and Materials Science-FB41, University of Groningen, University Medical Center Groningen, Groningen, A. Deusinglaan 1, 9713 AV Groningen, The Netherlands

**Keywords:** layered double hydroxides, Nile Red, SDS, hydrophobic, drug delivery

## Abstract

Layered double hydroxides (LDHs) have been extensively studied as drug delivery systems due to their favorable characteristics, including biocompatibility, high loading efficiency, and pH-responsive release. However, the current research predominantly focuses on LDHs as carriers for various anionic drugs, while there are only limited reports on LDHs as carriers for hydrophobic drugs. In this study, we successfully achieved the loading of a hydrophobic drug mimic, Nile red (NR), into LDHs using sodium dodecyl sulfate (SDS) as an intermediate storage medium. Furthermore, we optimized the experimental methods and varied the SDS/NR molar ratio to optimize this intercalation system. With an increase in the SDS/NR molar ratio from 2/1 to 32/1, the loading efficiency of LDH-SDS-NR for NR initially increased from 1.32% for LDH-SDS-NR_2/1 to 4.46% for LDH-SDS-NR_8/1. Then, the loading efficiency slightly decreased to 3.64% for LDH-SDS-NR_16.8/1, but then increased again to 6.31% for LDH-SDS-NR_32/1. We believe that the established method and the obtained results in this study broaden the application scope of LDHs as delivery systems for hydrophobic drugs and contribute to the further expansion of the application scope of LDHs.

## 1. Introduction

LDHs exhibit several appealing properties, including high anion exchange capacity [1], biodegradability [2], and biocompatibility [3], characteristics that make LDHs highly suitable for applications in drug delivery [4]. Previous studies have demonstrated the effectiveness of LDHs as carriers for a variety of therapeutic agents, such as anti-inflammatory drugs [5,6], antimicrobial agents [7,8], and adjuvants for DNA vaccines [9,10]. Although LDHs have been extensively studied as drug delivery systems, the majority of successfully loaded drug molecules are anionic due to the structural characteristics of LDHs. LDHs have positively charged layer plates, and negatively charged anions are required to balance the charge between layers to maintain their structural stability [11]. It is particularly challenging to load hydrophobic drugs, since the active state of hydrophobic molecules is often non-ionic and they are limitedly soluble in water. Therefore, it is not possible to use the commonly employed ion-exchange drug loading strategy to load them into LDH carriers.

To date, research on LDHs as hydrophobic drug delivery systems remains very limited, undoubtedly hindering the potential of LDHs as a universal drug delivery system. In previous studies, researchers have achieved the loading of the hydrophobic anticancer drug doxorubicin onto LDHs by surface adsorption through hydrogen bonding [12,13,14,15]. However, these studies did not address the structural bottleneck and technical challenges that make it difficult for LDHs to form intercalated structures with hydrophobic drug molecules. Furthermore, another study utilized micelles formed by the surfactant sodium cholate as intermediate carriers to load the hydrophobic drug camptothecin [16]. However, due to the spherical micelles requiring a significant amount of space within the LDH interlayer, the loading efficiency of camptothecin into LDHs was ultimately only 0.9%. It is worth noting that hydrophobic drugs are considerably represented in the field of pharmaceuticals, with the major drawback of poor solubility and hence bioavailability [17]. Many important drugs, including numerous anticancer agents [18], cardiovascular drugs [19], antibiotics [20], and central nervous system drugs [21], belong to the category of hydrophobic drugs. Therefore, researching LDHs as carriers for hydrophobic drugs contributes to the discovery of new, efficient, and safe carriers for the delivery of hydrophobic drugs, as well as to the universality of LDHs as drug carriers.

Exploring suitable intermediate storage media holds the potential to address the challenge of applying LDHs as an intercalation system for hydrophobic molecules. Sodium dodecyl sulfate (SDS), a common biocompatible anionic surfactant, is widely utilized as a coating for bio-nanomaterials to enhance their biocompatibility [22,23]. The SDS monomer possesses a hydrophilic, negatively charged headgroup and a hydrophobic tail [24], and SDS molecules will self-assemble into micelles when the concentration of SDS in the solution exceeds its critical micelle concentration (CMC) [25]. In past studies, SDS micelles have been investigated as drug delivery systems for hydrophobic drugs [26,27]. Therefore, the unique amphiphilic structure and biocompatibility of SDS make it an ideal candidate as an intermediate storage medium for the intercalation of hydrophobic drugs into LDHs, and its negatively charged headgroup can interact with the positively charged layer plates of LDHs to maintain the stability of the LDHs’ layered structure. However, although SDS has found its uses, other, more functional and even more biocompatible stabilizers should be investigated in the future. NR is used as a model compound in this study as it is a neutral molecule that contains an amine donor and a carbonyl acceptor [28], making it a versatile compound for the imaging of lipid droplets in tissues [29] and for protein characterization [30]. Moreover, due to its hydrophobicity, fluorescent properties, biocompatibility, and detectability, NR is widely used as a hydrophobic drug mimic in novel drug delivery systems [31,32,33,34].

In this study, NR was used as a hydrophobic drug mimic and SDS as an intermediate storage medium to facilitate the intercalation of NR into LDHs. SDS acts as an intermediate carrier for NR and forms SDS/NR complexes, which can then be loaded into the interlayer of LDHs through ion exchange. Additionally, we sought to optimize the hydrophobic drug intercalation system by adjusting the SDS/NR molar ratio, aiming to achieve the maximum loading efficiency of NR in LDHs. This research can serve as an important foundation for the use of LDHs in drug delivery systems for hydrophobic drugs with an enhanced loading capacity concerning the currently available LDH systems.

## 2. Experimental Section

### 2.1. Chemicals and Materials

All chemicals were utilized without additional purification. The layered double hydroxide (LDH) with the chemical formula Mg_4_Al_2_(OH)_12_CO_3_ 3.2·H_2_O was synthesized by the co-precipitation method, supplied by KISUMA CHEMICALS BV (Veendam, Netherlands), and used as received. Sodium dodecyl sulfate (SDS), Nile Red (NR), absolute methanol (CH_3_OH), hydrochloric acid (HCl), nitric acid (HNO_3_), sodium nitrate (NaNO_3_), and dichloromethane (CH_2_Cl_2_, DCM) were purchased from Sigma-Aldrich (Amsterdam, Netherlands). Milli-Q water with conductivity (18.2 × MΩ cm^−1^) was obtained with an Arium^®^ pro-DI Ultrapure purification system (Sartorius AG, Goettingen, Germany) and used in all experiments.

### 2.2. Synthesis of MgAl-NO_3_ LDH

The MgAl-NO_3_ LDH was synthesized by decarbonation and carbonate exchange according to the acid salt method described in our previous work [35]. Typically, 1 g of MgAl-CO_3_ LDH was added to a round-bottom flask containing 1 L of Milli-Q water, which was supplemented with 0.005 M HNO_3_ and 1.5M NaNO_3_. The suspensions were sealed and stirred mechanically at room temperature for 24 h. Subsequently, the resulting MgAl-NO_3_ LDH intercalated with NO_3_^−^ ions was separated by centrifugation, followed by two consecutive washes with Milli-Q water and absolute ethanol. The obtained product was then dried at 70 °C in an oven overnight.

### 2.3. Intercalation of NR onto LDH

NR (15.92 mg, 0.05 mmol) was dissolved in 20 mL DCM in a 250 mL conical flask and sealed under light-avoiding conditions by stirring until the NR was completely dissolved. Then, 0.1 mmol, 0.20 mmol, 0.40 mmol, 0.84 mmol, and 1.60 mmol of SDS were added to the separately prepared NR DCM solutions, respectively, and the solutions were sealed under light-avoiding conditions with stirring for 6 h to construct SDS/NR mixed systems with SDS/NR mole ratios of 2/1, 4/1, 8/1, 16.8/1, and 32/1. Afterward, the conical flask was left open to the air and stirring was continued overnight under light-shielding conditions to fully evaporate the DCM; finally, a thin film of SDS/NR was formed at the bottom of the conical flask. Subsequently, 100 mL Milli-Q water was added to the conical flask, and the SDS/NR thin film was redispersed in the aqueous solution using tip sonication (10 min) and stirring. The thoroughly ground LDH (20 mg) was added into the SDS/NR aqueous dispersion, and it was then stirred for 3 h, shielded from light, to allow SDS/NR to be fully loaded onto the LDH. The LDH-SDS-NR product was collected by centrifugation at 3700 rpm, and the product was then washed 3 times with absolute methanol and Milli-Q water alternately via redispersion and subsequent centrifugation at 3700 rpm. It was finally dried overnight in the oven at 70 °C. The resulting products were named LDH-SDS-NR_2/1, LDH-SDS-NR_4/1, LDH-SDS-NR_8/1, LDH-SDS-NR_16.8/1, and LDH-SDS-NR_32/1 according to the SDS/NR mole ratios.

### 2.4. Determination of the Loading Efficiency of LDH for NR

First, 2.5 mg of the LDH-SDS-NR nanohybrid was dissolved in 5M HCl (0.25 mL). After dilution with absolute methanol to 25 mL, the fluorescence emission spectra of NR were measured between 580 and 690 nm using a Microplate Fluorometer, with the excitation wavelength of 550 nm. Based on the obtained fluorescence emission spectra, the emission wavelength corresponding to the peak intensity of NR released from LDH-SDS-NR in methanol/0.1% 5M HCl solution was determined. Subsequently, the standard calibration curve for NR in methanol/0.1% 5M HCl solution was established at the determined emission wavelength and an excitation wavelength of 550 nm, and it was then used to calculate the loading amount of NR by LDH-SDS-NR. The loading efficiency of the LDH for NR was calculated using Equation (1). A one-way ANOVA test was performed using R (version 4.1.2).

(1) Loading efficiency of LDH-SDS-NRgg=amount of NR (g)amount of LDH-SDS-NR

### 2.5. Characterizations

The fluorescence analysis of Nile Red was performed using a Microplate Fluorometer (Thermo Scientific Fluoroskan, Amsterdam, Netherlands), with an excitation wavelength of 550 nm. Fourier transform infrared spectroscopy (FT-IR) spectra were obtained at a resolution of 4 cm^−1^ using the KBr pellet technique with a Cary 600 Series FT-IR Spectrometer (Agilent Technologies, Alexandria, VA, USA) over the range of 4000–400 cm^−1^. The crystalline analyses of LDHs were performed using X-ray diffraction (XRD) with a Bruker D-8 Advance Spectrometer (Bremen, Germany) equipped with a Cu X-ray tube, operated at 40 kV and 30 mA. LDH powders were scanned from 3° to 80° with a step size of 0.02°. 

## 3. Results

### 3.1. Preparation of LDH-SDS-NR Complexes

**Figure 1:**
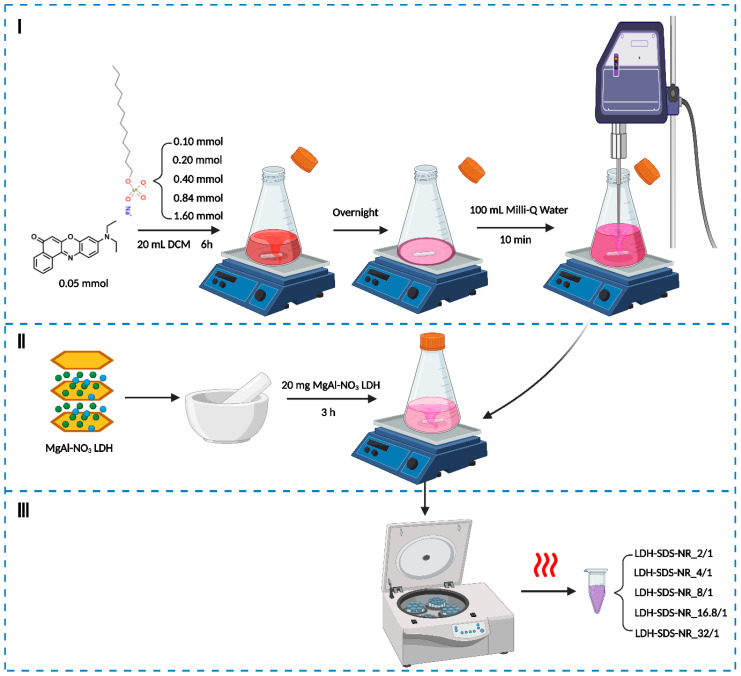
Schematic diagram of the construction process of LDH as an intercalation system for NR. Created with BioRender.com.

Figure 1 illustrates the strategy employed in this study to load the hydrophobic drug mimic NR into the LDHs. SDS was used as an intermediate storage medium for NR, and the overall construction process of LDH-SDS-NR was divided into three steps. Firstly, SDS/NR pre-encapsulation systems were prepared at different molar ratios of 2/1, 4/1, 8/1, 16.8/1, and 32/1, respectively. Subsequently, the ground MgAl-NO_3_ LDH was added to the SDS/NR mixed systems, which were redispersed in the aqueous solution using ultrasound. Finally, the LDH-SDS-NR intercalation systems under different molar ratios of SDS/NR were obtained by centrifugation, repeated washing via redispersion in water and methanol and subsequent centrifugation, and subsequent drying.

### 3.2. Characterization of LDH-SDS-NR

The MgAl-NO_3_ LDH used in this study was prepared following the method stated in our previous work, where MgAl-CO_3_ LDH was decarbonated using the acid salt method [35]. After decarbonation and carbonate exchange for NO_3_^−^, the FT-IR and XPS spectra of MgAl-NO3 LDH both exhibited new peaks corresponding to NO_3_^−^. Additionally, the XRD analysis revealed an increase in the interlayer distance of the MgAl-NO_3_ LDH from 0.757 nm (in MgAl-CO_3_ LDH) to 0.892 nm. Therefore, the successful preparation of the MgAl-NO_3_ LDH was confirmed by FT-IR, XPS, and XRD. The prepared and utilized MgAl-NO_3_ LDH is referred as the LDH for brevity.

**Figure 2:**
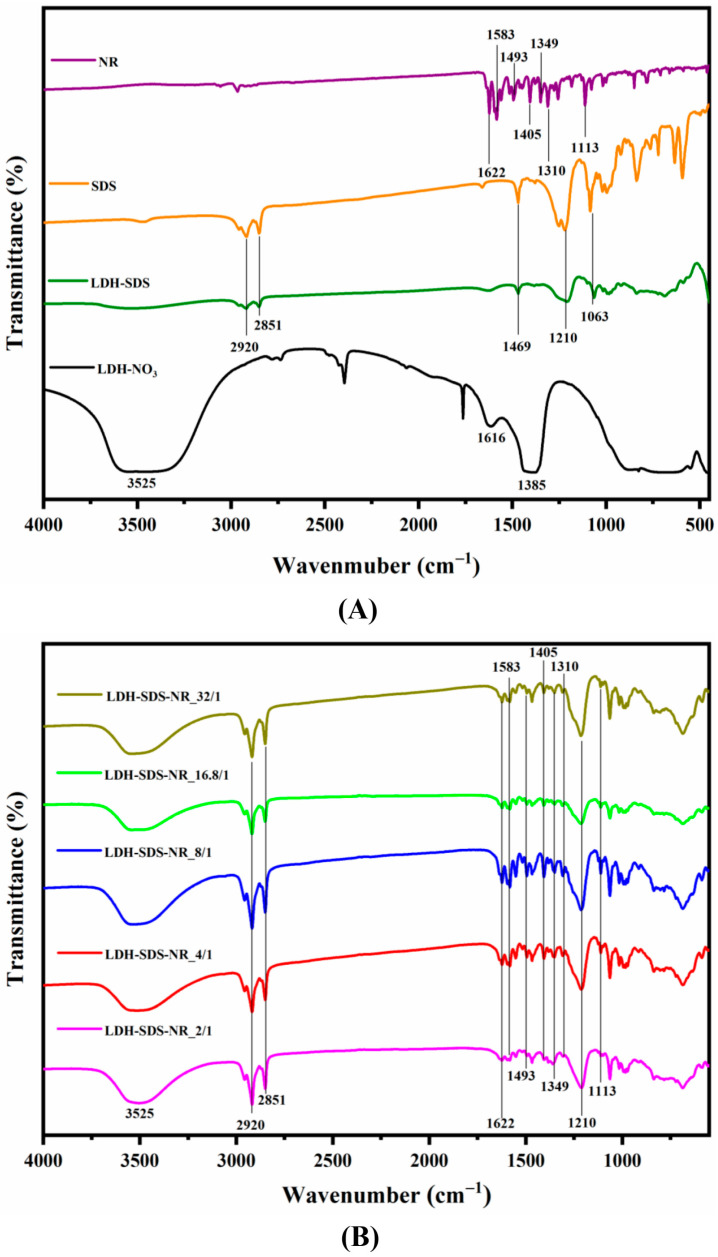
FT-IR spectra for LDH before (**A**) and after (**B**) NR loading.

The FT-IR spectra of the LDH before and after NR loading are presented in Figure 2, and the principal vibrational assignments of components of the sample are summarized in Table 1. The broad asymmetric signal at 3525 cm^−1^ is observed in all LDH samples and can be attributed to the stretching vibration of the hydroxyl groups present in the layers of the LDH, which are bound to magnesium and aluminum, as well as the hydroxyl groups of the interlayer molecules [36]. The shoulder observed at 1616 cm^−1^ can be attributed to the bending vibration of water molecules [37]. Furthermore, the peaks in the range of 400 cm^−1^ to 800 cm^−1^ are assigned to the skeleton vibration of Mg-OH and Al-OH bonds within the LDH layers [38]. Meanwhile, the peak observed at 1385 cm^−1^ in LDH-NO_3_ corresponds to its interlayer NO_3_^−^ ions [39]. Upon the intercalation of SDS, the LDH-SDS exhibits the emergence of new signals at 2920 cm^–1^ and 2851 cm^–1^, which can be assigned to the asymmetric stretching and symmetric stretching vibrations of CH_2_, respectively [40]. Furthermore, the appearance of new signals at 1210 cm^–1^ and 1063 cm^–1^ corresponds to the asymmetric and symmetric vibration peaks of S=O, respectively [41]. As shown in Figure 2B, the LDH exhibited characteristic peaks of NR after being loaded with SDS/NR at different molar ratios. In the 1622–1349 cm^−1^ region of all LDH-SDS-NR samples, the observed peaks correspond to the C-C stretching vibration of the aromatic ring in NR [42], while the peaks at 1310 cm^−1^ and 1113 cm^−1^ correspond to the stretching vibration of C-O in NR [43].

**Figure 3:**
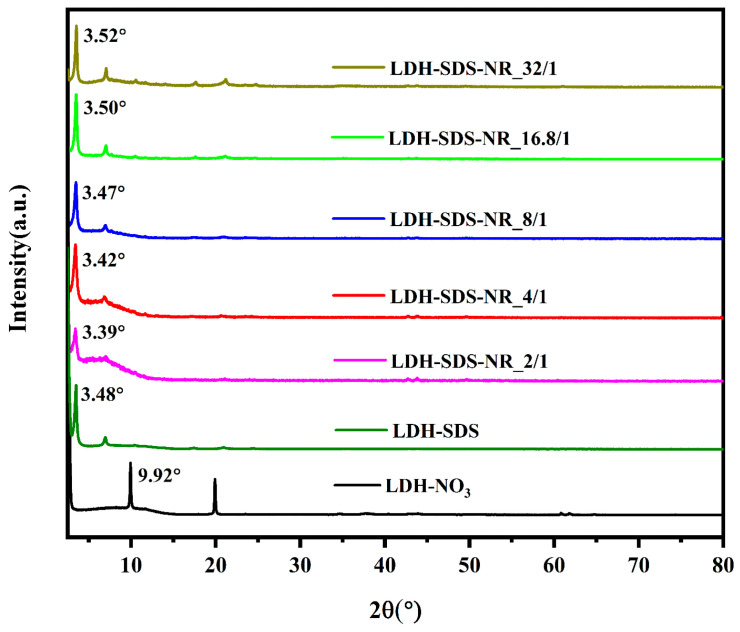
XRD patterns for LDH before and after NR loading.

The XRD patterns of the LDH before and after NR loading are presented in Figure 3. For the LDH samples, the first diffraction peak observed in the XRD pattern corresponds to the (003) crystal plane of the LDH [44], and the distance between LDH layers can be calculated from the diffraction angle of the (003) plane combined with Bragg’s equation (2dsinθ = nλ) [45]. Meanwhile, the thickness of the LDH layers is typically 0.48 nm; thus, the interlayer spacing within the LDH can be further determined. Moreover, the positions and angle information of the (003) plane for all samples are marked in Figure 3, and the angle information of the (003) plane and the interlayer distance of the LDHs calculated accordingly are summarized in Table 2. 

The interlayer spacing of LDH-NO_3_ is 0.41 nm. After loading with SDS, the interlayer spacing of LDH-SDS increases to 2.06 nm, while the chain length of the SDS molecules is 2.08 nm [46]. Therefore, for LDH-SDS, the SDS molecules are intercalated into the interlayers of the LDH, and the tilt angle between the SDS molecules and LDH layers is approximately 82° based on trigonometric calculations. Thus, the SDS molecules are arranged in a nearly perpendicular manner, with their long axis perpendicular to the LDH interlayers. After loading SDS/NR with a molar ratio of 2/1, the interlayer spacing of LDH-SDS-NR_2/1 increased to 2.13 nm, which was higher than the interlayer spacing of LDH-SDS (2.06 nm) and the chain length of SDS molecules (2.08 nm). As for NR, its length and width were approximately 1 nm and 0.7 nm, respectively [47]. The interlayer spacing of LDH-SDS-NR_2/1 (2.13 nm) can be attributed to the slightly larger size of the pre-encapsulated system SDS/NR, formed by the hydrophobic regions of SDS and NR. This results in the further enlargement of the interlayer spacing in the LDH upon the incorporation of SDS/NR. Although the interlayer spacing of LDH-SDS-NR_4/1 (2.10 nm) decreases slightly compared to LDH-SDS-NR_2/1 (2.13 nm), it is still higher than that of LDH-SDS (2.06 nm) and the length of the SDS molecule chains (2.08 nm). However, as the SDS/NR molar ratio was further increased to 8/1, the interlayer spacing of LDH-SDS-NR_8/1 decreased to 2.07 nm, which was slightly lower than the length of the SDS molecule chains but still slightly higher than the interlayer spacing of the control group LDH-SDS. It is worth noting that as the SDS/NR molar ratio was further increased to 16.8/1 and 32/1, the interlayer spacing of LDH-SDS-NR_16.8/1 and LDH-SDS-NR_32/1 decreased to 2.04 nm and 2.03 nm, respectively, which were slightly lower than the interlayer spacing of the control group LDH-SDS.

### 3.3. Drug Loading Efficiency of LDH-SDS-NR

**Figure 4:**
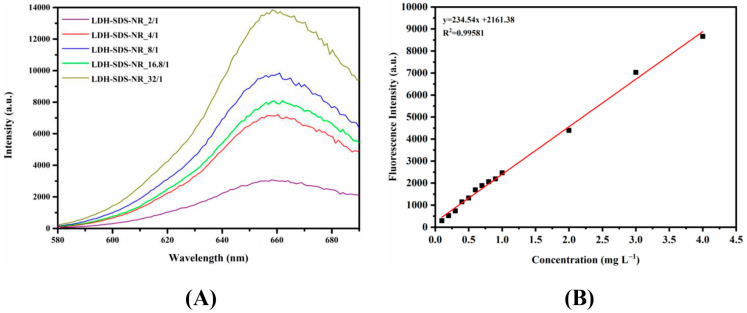
(**A**) Fluorescence emission spectra of loaded NR (λ_ex_ = 550 nm) from LDH-SDS-NR samples in methanol/0.1% 5M HCl. (**B**) Calibration curve of NR in methanol/0.1% 5M HCl with the excitation wavelength at 550 nm and emission wavelength at 658 nm.

The LDH was loaded with SDS/NR at different molar ratios and subsequently subjected to washing and drying to obtain the final LDH-SDS-NR samples. Then, the LDH-SDS-NR samples were dissolved in 5M HCl to release all of the loaded NR from the LDH, followed by dilution with absolute methanol. Then, 5M HCl was used to disrupt the layered structure of the LDHs, allowing the complete release of the loaded drug, thus accurately determining the drug loading efficiency of the LDHs. The fluorescence emission spectra of loaded NR (λ_ex_ = 550 nm) from different LDH-SDS-NR samples in methanol/0.1% 5M HCl were measured between 580 and 690 nm using a Microplate Fluorometer. Based on the obtained fluorescence emission spectra (Figure 4A), the emission wavelength corresponding to the peak intensity of NR released from LDH-SDS-NR in methanol/0.1% 5M HCl solution was observed at 658 nm. Subsequently, the standard calibration curve for NR in methanol/0.1% 5M HCl solution was established with the excitation wavelength at 550 nm and emission wavelength at 658 nm, and it was then used to calculate the loading efficiency of NR into the LDH. As shown in Table 3, with the increase in the molar ratio of SDS/NR from 2/1 to 8/1, the loading efficiency of the LDH towards NR increased from 1.32% for LDH-SDS-NR_2/1 to 3.24% for LDH-SDS-NR_4/1 and 4.46% for LDH-SDS-NR_8/1, respectively. However, when the molar ratio of SDS/NR reached 16.8/1, the loading efficiency of LDH-SDS-NR_16.8/1 towards NR decreased compared to LDH-SDS-NR_8/1 (4.46%) and was measured at 3.64%. Interestingly, when the molar ratio of SDS/NR was further increased to 32/1, the loading efficiency of LDH-SDS-NR_32/1 reached 6.31%.

## 4. Discussion

In this study, we aimed to utilize the intermediate storage medium, SDS, to achieve the loading of a hydrophobic drug mimic, NR, into LDHs. SDS is a commonly used surfactant [48], and when the concentration of SDS reaches its critical micelle concentration (CMC), the SDS molecules self-assemble to form micellar structures. It has been reported in the literature that the CMC value of SDS is 8.4 mM [49]. It is worth noting that micelle formation is a multi-step progressive process. In a previous study, Cui et al. [50] investigated the process and mechanism of micelle formation of SDS in an aqueous solution using ^1^H NMR and NMR self-diffusion experiments. The results of the study showed that the micellization of SDS is a multi-step process, where the SDS molecules in a solution first associate to form small aggregates at concentrations below the CMC. As the SDS concentration increases, larger aggregates are formed, and micelle formation begins at the CMC, but pre-micellar aggregates still coexist. Finally, at concentrations above the CMC, more pre-micellar aggregates of SDS grow into micelles, leading to an increased proportion of micelles in the solution, although pre-micellar aggregates and SDS monomers still exist.

**Figure 5:**
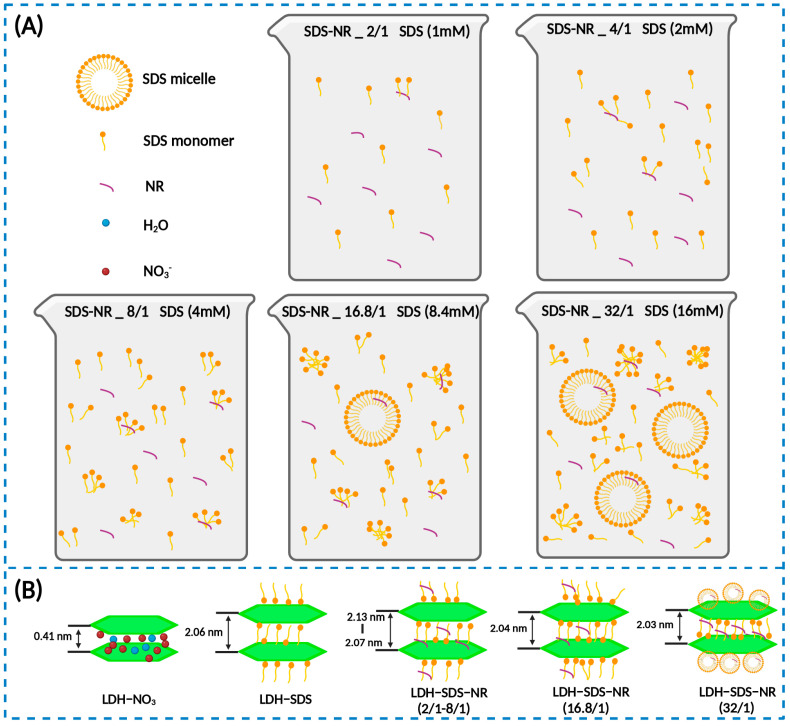
The schematic diagram of the effects of different molar ratios of SDS/NR feed on the dispersion state of SDS and the SDS/NR encapsulation system (**A**), as well as the loading behaviors of LDHs towards SDS/NR with different molar ratios (**B**). Created with BioRender.com.

In our work, we varied the amount of SDS while keeping the dosage of NR constant to alter the initial molar ratio of SDS/NR, in order to explore and optimize the loading system of LDHs for hydrophobic agents. As the molar ratio of SDS/NR increased from 2/1 to 32/1, the corresponding molar concentrations of SDS in the system were 1 mM (SDS-NR_2/1), 2 mM (SDS-NR_4/1), 4 mM (SDS-NR_8/1), 8.4 mM (SDS-NR_16.8/1), and 16 mM (SDS-NR_32/1). Therefore, in the selection of the SDS concentration, a range below the CMC, the CMC value itself, and a concentration above the CMC were considered. The schematic diagram of the effects of different molar ratios of SDS/NR feed on the dispersion state of SDS and the SDS/NR mixed system is shown in Figure 5A. The loading behaviors of LDHs towards SDS/NR with different molar ratios are shown in Figure 5B. As the concentration of SDS was below the CMC of SDS (1 mM–4 mM), the loading efficiency of LDHs for SDS/NR increases with the increase in the SDS concentration. When the molar ratio of SDS/NR is 2/1, there are fewer SDS aggregates in the system, resulting in the lower encapsulation efficiency of NR and relatively low loading efficiency, which is 1.32%. However, as the SDS concentration increases, more SDS aggregates are formed in the system, leading to improved efficiency in NR encapsulation. Consequently, the loading efficiencies of NR for LDH-SDS-NR_4/1 (3.24%) and LDH-SDS-NR_8/1 (4.46%) also increase accordingly. It is worth noting that the interlayer spacing of LDH-SDS-NR decreases as the SDS concentration increases, from 2.13 nm for LDH-SDS-NR_2/1 to 2.10 nm for LDH-SDS-NR_4/1 and 2.07 nm for LDH-SDS-NR_8/1. This could be attributed to the increased concentration of SDS, resulting in a larger number of SDS molecules intercalating into the interlayer spaces of LDHs. The repulsive forces between negatively charged SDS molecules may cause a slight alteration in their orientation, thereby affecting the inner spacing of LDHs. Within the range of SDS-NR molar ratios from 2/1 to 8/1, the interlayer spacing of LDH-SDS-NR is higher than that of the control group, LDH-SDS, without NR loading (2.06 nm). Moreover, by combining the FT-IR spectra of LDH before and after NR loading, we can infer that the LDH-SDS-NR_2/1, LDH-SDS-NR_4/1, and LDH-SDS-8/1 systems successfully achieved NR loading, and NR-intercalated LDH structures existed within the systems.

When the SDS concentration in the system reaches the CMC, SDS pre-micellar aggregates begin to form, followed by SDS micelles. According to previous studies, the diameter of SDS is approximately 3–4 nm [51,52,53]. Although the formation of micelles and pre-micellar SDS aggregates further enhances the encapsulation efficiency of NR, their larger size makes it difficult for them to undergo ion exchange with LDHs to form intercalation structures. Therefore, due to the existence of fewer SDS/NR complexes of favorable sizes, the loading efficiency of LDH-SDS-NR_16.8/1 (3.64%) decreases compared to LDH-SDS-NR_8/1 (4.46%). Nevertheless, when the SDS concentration exceeds its CMC value, the quantity of SDS/NR complexes with favorable sizes for intercalation into LDHs also increases, leading to an increase in the loading efficiency of 6.31% (LDH-SDS-NR_32/1). Additionally, when the SDS concentration is at or above the CMC, the interlayer spacing of LDH-SDS-NR_16.8/1 and LDH-SDS-NR_32/1 further decreases to 2.04 nm and 2.03 nm, respectively. It is noteworthy that the interlayer spacing of both LDH-SDS-NR_16.8/1 and LDH-SDS-NR_32/1 is lower than that of the control group LDH-SDS (2.06 nm). In systems where the concentration of SDS is above or at its CMC, SDS monomers and various-sized SDS aggregates still exist alongside SDS micelles [50]. Moreover, through the complete washing of the samples, the non-intercalated and surface-adsorbed NR was likely completely removed. The rigorous washing, which was done with methanol, could also have had an additional effect on the final measured loading efficiency. While the loading efficiency of the hydrophobic drug intercalation system based on LDHs established in this study has been improved compared to previous research findings, it should be noted that the actual loading efficiency of this hydrophobic drug carrier system may still be significantly higher than the determined values. During the purification process, extensive washing with water and methanol was performed to thoroughly remove unloaded NR. However, this process might have inevitably resulted in the release of the NR intercalated within the LDH layers as well. Therefore, further optimization of the purification process of this LDH-based hydrophobic drug intercalation system could further identify an even greater enhancement in its loading efficiency.

In two previous research works, researchers attempted to utilize another surfactant, sodium cholate (SC), as an intermediate storage medium for the intercalation of hydrophobic agents into LDHs. The CMC value of SC is 16 mM [54], and both of these two studies directly set the concentration of SC above the CMC. In one study, an SC system with a concentration of 17.43 mM was used in an aqueous solution, followed by the sequential addition of the hydrophobic drug camptothecin (CPT) and LDHs. The resulting LDH-SC-CPT achieved loading efficiency of only 0.9% for CPT [16]. In another study, an SC system with a concentration of 30.22 mM was used in Tris buffer, followed by the sequential addition of the hydrophobic drug gramicidin and LDHs, achieving loading efficiency of 2.2% for gramicidin [55]. It is evident that, in our study, in systems where the SDS concentration was close to or above its CMC, the loading efficiencies of LDH-SDS-NR_16.8/1 (3.64%) and LDH-SDS-NR_32/1 (6.31%) for hydrophobic agents were higher than those reported for LDH-SC-CPT (0.9%) and LDH-SC-gramicidin (2.2%) in the aforementioned studies. Moreover, LDH-SDS-NR_8/1 obtained with the concentration of SDS below its CMC still had higher loading efficiency for NR (4.46%) compared to LDH-SDS-NR_16.8/1 (3.64%). It needs to be recognized that the micellar system is not necessarily the optimal system to achieve the LDH intercalation of hydrophobic drugs through an intermediate storage medium. This is because micelles themselves have a relatively large size, which makes it difficult for larger micellar systems to undergo ion exchange with LDHs. Moreover, even if intercalation is achieved, it may occupy a large space within the LDH interlayers, thereby affecting the loading efficiency. Additionally, the aforementioned studies both established a micellar system first, followed by the addition of a hydrophobic drug. On one hand, adding the hydrophobic agents after the complete formation of the micellar system may make them less accessible for encapsulation by micelles or pre-micellar aggregates. On the other hand, the solvents used in these two studies could not fully dissolve the hydrophobic agents, which could also have limited the encapsulation of the drugs by SC and ultimately affected the loading efficiency of hydrophobic drugs into the LDH. 

In this study, we first established a highly compatible SDS/NR mixed system in an organic solvent that was suitable for NR. Subsequently, we dispersed this system in an aqueous solution to achieve the LDH loading of SDS/NR. Additionally, for the first time, we attempted to optimize the drug loading system of LDHs for the hydrophobic drug mimic NR by varying the molar ratio of SDS/NR. These results provide important insights for breakthroughs in the LDH-mediated delivery of hydrophobic drugs. Moreover, in future work, the further analysis of the structure of LDH-SDS-NR is required. For instance, small-angle X-ray diffraction can be employed to investigate the arrangement of SDS/NR within the LDH layers and provide direct evidence for the intercalation structure of LDH-SDS-NR at concentrations above the CMC of SDS. Additionally, it is necessary to conduct further studies on drug release in this hydrophobic drug loading system, which will not only contribute to a better understanding of the mechanisms involved in hydrophobic drug loading but also facilitate its broader application. In general, the methodology established in this study provides a crucial reference and lays a solid foundation for research involving the use of LDHs in the delivery of hydrophobic drugs. Compared to other studies, our approach effectively enhanced the loading efficiency of hydrophobic drugs onto LDHs. Simultaneously, the system developed in this research contributes to broadening the application scope of LDHs, rendering them universally suitable for various types of loaded drugs, rather than being confined solely to hydrophilic drugs. Furthermore, there is great potential for the further exploration of LDHs as the next generation of novel, efficient, and safe hydrophobic drug delivery systems, thereby overcoming the challenges associated with the effective delivery of hydrophobic drugs in the field of pharmaceutical sciences.

## 5. Conclusions

Given the numerous advantages of LDHs as drug carriers and the limited research on their use as carriers for hydrophobic drugs, establishing a strategy for the use of LDHs as intercalation carriers for hydrophobic drugs is of significant importance. In this study, SDS acted as an intermediate carrier for NR (a hydrophobic drug mimic) and formed SDS/NR complexes, which could then be loaded into the interlayers of LDHs through ion exchange. Moreover, the LDH loading system for hydrophobic drugs was optimized by varying the SDS/NR molar ratio. The results revealed that as the SDS/NR molar ratio increased from 2/1 to 8/1, the loading efficiency of LDH-SDS-NR for NR improved from 1.32% (LDH-SDS-NR_2/1) to 4.46% (LDH-SDS-NR_8/1). However, due to the existence of fewer SDS/NR complexes of favorable sizes, the loading efficiency of LDH-SDS-NR_16.8/1 decreased to 3.64%. Nevertheless, when the SDS concentration exceeds its CMC value, the quantity of SDS/NR complexes with favorable sizes for intercalation into LDHs also increases, leading to an increase in the loading efficiency of 6.31% (LDH-SDS-NR_32/1). In future work, the further analysis of the internal structures of LDH-SDS-NR is required. Additionally, studies on drug release are needed for a comprehensive understanding of this LDH-based system for hydrophobic drugs. Certainly, the strategy developed and the results obtained in this study will serve as a crucial reference and establish a solid foundation for research on the use of LDHs as delivery systems for hydrophobic drugs.

## Figures and Tables

**Table 1:** Principal vibrational assignments of components of the samples.

Band Position (cm^−1^)	Assignment ^a^
3525	νa (OH)
2920	νa (CH_2_)
2851	νs (CH_2_)
1622, 1583, 1493, 1405, 1349	ν (C-C)
1616	δ (H_2_O)
1469	(CH_2_) shear
1385	ν (NO_3_^−^)
1310, 1113	ν (C-O)
1210	a (S=O)
1063	s (S=O)

^a^ ν: stretching; δ: bending; a: asymmetric; s: symmetric.

**Table 2:** Comparison of structural parameters for LDH before and after NR loading from XRD.

Samples	2θ (°)	Interlayer Spacing (nm)
LDH-NO3	9.92	0.41
LDH-SDS	3.48	2.06
LDH-SDS-NR_2/1	3.39	2.13
LDH-SDS-NR_4/1	3.42	2.10
LDH-SDS-NR_8/1	3.47	2.07
LDH-SDS-NR_16.8/1	3.50	2.04
LDH-SDS-NR_32/1	3.52	2.03

**Table 3:** The loading efficiency of LDH for NR at different SDS/NR molar ratios *.

Samples	Loading Efficiency (%)
LDH-SDS-NR_2/1	1.32 ± 0.10 a
LDH-SDS-NR_4/1	3.24 ± 0.11 b
LDH-SDS-NR_8/1	4.46 ± 0.10 d
LDH-SDS-NR_16.8/1	3.64 ± 0.07 c
LDH-SDS-NR_32/1	6.31 ± 0.05 e

* The lowercase letters indicate significant differences between groups (one-way ANOVA with Tukey HSD test, *p* < 0.05). Each value represents the mean ± SD (n = 3).

## Data Availability

The data presented in this study are available upon request from the corresponding author. The data are not publicly available due to privacy.
